# The Identification of the Dead

**Published:** 1897-11

**Authors:** 


### THE IDENTIFICATION OF THE DEAD.

There has been renewed interest in this subject on the part of the general public since the Charity Bazar fire in Paris, and Dr. Marcel Baudouin contributes some notable points in an article published in the *Progres Medical* for October 2d. In such catastrophes, he remarks, the body is almost entirely consumed, and the greatest amount of information as to the identity of the person, with the exception of articles found about the remains, is to be derived from the skeleton, and especially from the teeth, which often escape the violence of the fire. When, says M. Baudouin, the bodies are those of well-to-do persons, and above all those of people in high life, each individual may be assumed to have a dental apparatus which of itself possesses very characteristic features. In the first place, the mere form of the jaws is a guide to the sex and the age, and the same is true of the number of teeth that remain. But, more than all this, each individual tooth is a valuable piece of evidence. If it is sound, it may, by its whiteness, its form, etc., lead to the recognition of sex and social condition; if it is diseased and has been subjected to treatment, it may prove the means of a positive identification. In the case of the body of the Duchess d'Alencon, who perished in the Bazar fire, says M. Baudouin, her dentist identified her jaw in the charred remains of a skull by examining it in the light of notes in his case-book. In this connection, some of our readers will doubtless recall the Parkman murder case, which occurred in Boston many years ago. The murderer was a professor of chemistry, and he disposed of his victim's body by consuming it entirely, as he supposed, in his furnace. But Parkman had been provided with artificial teeth, and those teeth escaped destruction. The dentist who had made them swore to their identity, and it was mainly on his testimony that Professor Webster, a fearfully tempted man, was convicted.

In identifying the trunk of a decapitated body, says M. Baudouin, there are certain signs that may be of great service. He cites the case of the supposed corpse of Mme. de Luppe, who was known to have undergone the operation of ovariotomy. No trace of an incision was found on the abdomen. Pure anthropometry, says M. Baudouin, will not always suffice for the identification of a dead body, and it is the purpose of his article to urge closer co-operation between that science, medicine, and the police system. Sharp as the police, the experts, and the courts are, he declares, they still let slip many data—documents, written or unwritten, he calls them—that might be most useful. Among them he mentions the intellectual peculiarities of the individual, but on this point he refrains from enlarging, saying that to go into the details would lead him too far. The person's handwriting is next spoken of. Henceforth, says M. Baudouin, it should always be the subject of careful investigation. He avows that he is no graphologist, but says that for him the handwriting is as expressive as any man's face. It is useless to say, he adds, that chirography may be simulated, for so, too, may facial expression. While he does not accept the handwriting as an indisputable index of character, he does regard it as a valuable sign of identity, quite as much as the prints of a man's bare feet, or Galton's lines on the thumb, or Bertillon's measurements of the bones.—*N. Y. Medical Journal.*

